# Valorisation of Sub-Products from Pyrolysis of Carbon Fibre-Reinforced Plastic Waste: Catalytic Recovery of Chemicals from Liquid and Gas Phases

**DOI:** 10.3390/polym16050580

**Published:** 2024-02-21

**Authors:** Esther Acha, Naia Gastelu, Alexander Lopez-Urionabarrenechea, Blanca María Caballero

**Affiliations:** Chemical and Environmental Engineering Department, Faculty of Engineering of Bilbao, University of the Basque Country (UPV/EHU), Plaza Ingeniero Torres Quevedo, 1, 48013 Bilbao, Spain; naia.gastelu@ehu.eus (N.G.); alex.lopez@ehu.eus (A.L.-U.); blancamaria.caballero@ehu.eus (B.M.C.)

**Keywords:** pyrolysis, carbon fibre-reinforced plastics, recycling, catalyst, hydrogen

## Abstract

Waste carbon fibre-reinforced plastics were recycled by pyrolysis followed by a thermo-catalytic treatment in order to achieve both fibre and resin recovery. The conventional pyrolysis of this waste produced unusable gas and hazardous liquid streams, which made necessary the treatment of the pyrolysis vapours. In this work, the vapours generated from pyrolysis were valorised thermochemically. The thermal treatment of the pyrolysis vapours was performed at 700 °C, 800 °C and 900 °C, and the catalytic treatment was tested at 700 °C and 800 °C with two Ni-based catalysts, one commercial and one homemade over a non-conventional olivine support. The catalysts were deeply characterised, and both had low surface area (99 m^2^/g and 4 m^2^/g, respectively) with low metal dispersion. The thermal treatment of the pyrolysis vapours at 900 °C produced high gas quantity (6.8 wt%) and quality (95.5 vol% syngas) along with lower liquid quantity (13.3 wt%) and low hazardous liquid (92.1 area% water). The Ni–olivine catalyst at the lowest temperature, 700 °C, allowed us to obtain good gas results (100% syngas), but the liquid was not as good (only 58.4 area% was water). On the other hand, the Ni commercial catalyst at 800 °C improved both the gas and liquid phases, producing 6.4 wt% of gas with 93 vol% of syngas and 13.6 wt% of liquid phase with a 97.5 area% of water. The main reaction mechanisms observed in the treatment of pyrolysis vapours were cracking, dry and wet reforming and the Boudouard reaction.

## 1. Introduction

Fibre-reinforced plastics were a revolution a few decades ago due to their exceptional weight/mechanical properties ratio. This meant that they were initially used in sectors where the energy balance due to weight was very critical and which could afford to invest heavily in newly developed materials. This was the case, for example, in aeronautics, automotive racing, and high-performance parts or equipment in the 1990s or 2000s [1]. Gradually, their use was extended to wind turbines, automotive, sports and boats, among others. Nowadays, these materials are present in a large number of different types of structures, consumer goods or objects, to a greater or lesser extent in their composition. As an example, in 1975, the A300 aircraft of Airbus had around 5% of fibre-reinforced plastics, while the A350 in 2010 had more than 50% [2]. The future storage and use of hydrogen in cars or aircrafts will also ramp up the production of fibre-reinforced plastics worldwide in the long run [3].

During these years of massive production of fibre-reinforced plastics, research was still focused solely on designing and manufacturing these composite materials. In other words, those aircraft or wind turbines that were manufactured with fibre composite materials reached the end of their useful life without there existing any viable technological solution for their recycling. Today, this situation is gradually being reversed. Research into their recovery and recycling has been progressing for years, but no technology has yet been established in a massive and global way. It is essential to continue researching the recycling of these composite materials, which can have a highly variable composition. Factors affecting composition are: the type and amount of fibre and resin, the additives they contain, the possible mixture of carbon and glass fibre, the arrangement of the fibre, the variable size and volume of the part to be recycled, the possible contamination by paints or other coatings, the content of cables and other materials, etc. [4]. This material is, therefore, being accumulated in landfills or dumps or, at most, some of it is sent to incinerators where only energy is recovered. Moreover, at the time of manufacture, the materials that are employed are known, but over the years, when it becomes a waste, that information is lost and all the fibre residues are collected together, separated, at best, into glass and carbon fibre.

The ideal recycling of this type of fibre composite material would allow for the fibres to be recovered, with a greater or lesser loss of properties depending on the technology and conditions used for recycling, as well as being able to take advantage of the material that is intrinsic in the resin or other materials that make it up. Although recovered fibres may have poorer properties than virgin fibres, it will always be possible to reintroduce them into a production cycle and, thus, promote the circular economy due to the wide variety of manufacturing processes and types of materials in which they can be used [5,6]. Some of the possible uses for the recycled fibres are: cement-based materials, lithium-ion battery materials, thermoelectric materials, and other functional fields (filling material) [7].

Over the last decade, several routes for the recycling of fibre-reinforced composites have been studied. There are three main recycling methods [8]: (I) Mechanical, which involves shredding the material into small pieces to try to incorporate the shredding into a future composite material to be manufactured. (II) Chemical, such as solvolysis, which focus on releasing the carbon fibre by dissolving the resin. It is a complex procedure, which generates a residual liquid stream, without the possibility of taking advantage of the resin, is very sensitive to the composition of the composite material, and is difficult to apply to complex and heterogeneous waste. (III) The third method is thermal, in which pyrolysis is located. Among those options, the most versatile and with highest TRL (Technology Readiness Level) achieved so far is pyrolysis [9]. This treatment allows for great flexibility in terms of the waste fed. In addition, the operating parameters can be modified according to the desired output products, and the process itself can be somehow adjusted to the type of waste being fed in each specific moment. There are facilities where fibres are recovered by pyrolysis of end-of-life waste, such as, for example, Gen 2 Carbon (UK) or Carbon Rivers (USA), so this part is already industrialised, and in principle, the development and research needs are lower. It is important to note that the pyrolysis of these materials generates three phases as products: a solid phase, from which the fibres are recovered, and a liquid phase and a gaseous phase, whose best recovery route has not yet been decided. These two streams (liquid and gas), in the ideal and maximum case, represent the equivalent weight of resin in the waste to be treated, which could be more than half of the total weight, so they are important streams to take into account in the overall process of recycling carbon fibre composite materials.

However, the recovery of all the material that is not fibre (resins, paints, contaminations, additives, etc.) is much more complex and still requires a lot of development. This recovery needs to be given a boost, so that end-of-life recycling of fibre-reinforced plastics can finally be implemented with maximum material recovery, maximum circularity and maximum cost-effectiveness. In the recycling of fibre-reinforced plastics by pyrolysis, the recovery of non-fibrous material can focus on different targets. Among others, hydrogen, syngas, raw materials for chemical manufacturers or refineries and gas/liquid to be used as an internal energy supply in the plant itself can be produced [10].

In the case of the work presented in this article, which focuses on the resin valorisation of carbon fibre-reinforced plastics, experimental tests are carried out with pre-preg, which is homogeneous in its composition. This is an essential first step to be able to analyse different resin valorisation options, eliminating variability in results due to the heterogeneity of the material studied. The previous work of the research team with this pre-preg was focused mainly on increasing the production of gases in addition to recycled fibre [11,12,13]; however, it has been observed that the production and management of liquids is also an important issue. Therefore, success will be achieved with a high production of high-quality gas (high hydrogen content or synthesis gas) together with a low production of low-hazard liquid. This liquid should have a composition that is as simplified as possible, which in an ideal case, would allow for some chemicals to be recovered or, if not, would allow for it to be treated as wastewater that after minor treatment could be discharged together with the rest of the industrial water to the sewer. This would be a great step forward, since without specific treatment this liquid must be treated as a hazardous liquid, to be stored in the facilities and removed by an authorised hazardous waste manager; thus, it entails a high environmental, social and economic impact.

The novelty of this work is the incorporation of a treatment in the recycling process for the vapours generated from the pyrolysis of the pre-preg, which allows for thermal cracking to be carried out; in addition, if a catalyst is incorporated in this reactor, it gives the option of carrying out a thermo-catalytic process. Through this stage, it is possible to transform the larger molecules that tend to condense at the exit of the pyrolysis reactor, generating a liquid phase with a complex and dangerous composition, into lighter compounds with high added value in the gas. For this purpose, two nickel-based catalysts have been used, which are widely studied reforming catalysts as they have an optimal balance between cost, availability on the planet, activity and stability compared to noble metals [14,15,16]. One of the catalysts was a commercially available Ni catalyst for reforming reactions. The other catalyst was a homemade Ni-based catalyst with a cobalt addition, which promotes C-C bond rupture [17], and a Pd addition, which can improve the stability of the catalyst, decreasing the tendency of Ni to form carbon [18,19]. This homemade catalyst was supported over a non-conventional olivine support. This natural substance, which is readily available and cost-effective, is characterised by simple production and operational processes compared to synthetic supports [20,21].

The objective of this work was to achieve for the first time, to the best of the authors’ knowledge, a complete and integral recycling process of carbon fibre composite waste, paying attention, in this case, to the recovery of the resin. To achieve this, a pyrolysis process was performed to the waste with an in-line thermo-catalytic treatment of the vapours produced to obtain a high quantity of high-quality gas and a low quantity of a low-hazard liquid. Thermal and thermo-catalytic treatment were compared, and the optimum operating conditions were selected from an overall point of view of the recycling process.

## 2. Materials and Methods

### 2.1. Carbon Fibre Composite

In order to carry out the experimental work shown in this paper, an expired pre-preg from manufacturing activities in the aeronautical industry was used, manufactured by Hexcel and commercially named M21. Specifically, it is a composite material of epoxy resin in a proportion of 34 wt% and unidirectional carbon fibres with a 66 wt%. These pre-impregnated rolls are used in automatic wrappers according to the automatic tape laying technique (ATL) and are considered waste (out of date) after approximately one month out of low-temperature storage conditions or after one year from manufacture. Both the physical and chemical properties of this material have been described and discussed in detail by the authors previously [11].

### 2.2. Catalysts and Reactor Bed Material

Two different catalysts were used for the treatment of pyrolysis vapours: a commercial nickel-based catalyst (Ni commercial) from the company Katalco Johnson Matthey (London, UK) and a catalyst over olivine prepared in the laboratory (Ni–olivine). The commercial catalyst had the following composition: 50 wt% NiO, 4 wt% SiO_2_ and 1.5 wt% Cr_2_O_3_. The catalyst supported on olivine ore, which was magnesium iron nesosilicate, was prepared using olivine supplied by the company Ilarduya y Cía (Bilbao, Spain), composed of 48 wt% MgO, 41 wt% SiO_2_ and 8 wt% Fe_2_O_3_. The wet impregnation method was used for the preparation of the olivine-based catalyst by preheating the support at 700 °C for 4 h before impregnation. The theoretical composition of the prepared catalyst was 30 wt% Ni, 10 wt% Co and 1 wt% Pd. The precursors of the metals were Ni (NO_3_)_2_·6H_2_O (99.99 wt%; Sigma-Aldrich, St. Louis, MO, USA), Co(Cl)_2_·6H_2_O (98 wt%; Sigma-Aldrich) and Pd(NO_3_)_2_·2H_2_O (40 wt% Pd, Sigma-Aldrich). To obtain the above catalyst composition, the appropriate amounts of supports and metal precursors were mixed in 10 mL of distilled water per gram of support. After mixing overnight, around 12 h, at 45 °C in the rotary evaporator under vacuum, the water was removed. It was then placed in the oven at 110 °C overnight, around 12 h, to ensure that it was completely dry. Both catalysts were calcined before use at 700 °C for 4 h under the influence of atmospheric air, using a heating rate of 3 °C/min.

The catalysts were introduced into the tubular reactor in the form of particles in size 0.42–0.5 mm, so that the ratio between the internal diameter of the reactor and the particle diameter was greater than 10, thus avoiding the formation of vial lanes adjacent to the wall [22]. The catalyst was placed in the middle part of the tubular reactor, where the desired operating temperature was ensured, and mixed with CSi of the same particle size. The rest of the tubular reactor, or the complete reactor in the case of the tests in which no catalyst was used, was filled with a residual refractory brick (manufacturing rejects) of high alumina supplied by Beroa S.L. (Bilbao, Spain). This mixture ensured a best thermal dispersion inside the tubular reactor.

### 2.3. Pyrolysis Installation and Experiments

The experimental pyrolysis setup consisted of two reactors in series followed by a condensation train and a gas collection area. A detailed diagram of the lab-scale installation is given in previous work from the authors [12]. The first reactor was a tank reactor, where approximately 100 g of the residual composite material to be pyrolysed (3.5 L) was introduced. The second reactor was tubular, and here, both thermal and catalytic treatment of the vapours were carried out (50 cm long and 2.5 cm in diameter). The outlet of the reactors was connected to a condensation train consisting of three condensers (one at ambient temperature and two of them with water circulation cooling), where the condensable compounds were collected in liquid phase. All uncondensed compounds passed through an activated carbon filter (for safety of the gas analysis system) and were then collected in Tedlar bags for their later analysis.

Several tests were carried out modifying the conditions of the tubular reactor (temperature and catalyst). In all the tests, the batch reactor was heated up to 500 °C with a heating rate of 3 °C/min. Once 500 °C was reached, the process was shut down and the pyrolysis furnace was allowed to cool down. During the pyrolysis process, no N_2_ flows were fed into the pilot plant. The influence in the tubular reactor of the following parameters was studied: (I) tests without vapour treatment (for comparison), consisting of an assembly without tubular reactor; (II) tests carried out to analyse the effect of the thermal treatment (700, 800, 900 °C) and (III) tests to analyse the effect of the thermo-catalytic treatment with the two catalysts and two temperatures (700 Ni commercial, 700 Ni–olivine, 800 Ni commercial).

### 2.4. Analytical Techniques

The collected amount of solid and liquid after the pyrolysis experiments was calculated by initial and final weight difference, and the gas yield was calculated by difference to 100. These values are shown in pyrolysis yield mode, with respect to the initial total mass of material fed into the tank reactor. The collected solid was not characterised, as the pyrolysis conditions in the tank reactor, where the solid is placed, were not changed in this work and the properties have been already published by the authors [12,23,24]. In these studies, reclaimed clean carbon fibres, with mechanical properties within commercial values, were obtained after controlled oxidation of the solid remaining after the pyrolysis step. The liquid and gas collected were analysed by different techniques, which are described below.

The composition of the condensed liquid collected in the condensation train was determined by gas chromatography-mass spectrometry (GC-MS) (AGILENT 6890, AGILENT 5973, Santa Clara CA, USA). Compounds were considered well identified when the software library showed at least an 85% quality value in the match parameter. If the match was lower, that compound was classified as “unidentified”. Compound values are given in area%, and in case the area% value is less than 3%, it is counted as “other”.

The gases collected after condensation, after passing through the activated carbon filter to protect the analytical equipment, were analysed with a GC (AGILENT 7890A, Santa Clara CA, USA) coupled with a thermal conductivity detector (TCD) and flame ionisation detector (FID). The chromatograph had two columns (HP-MOLESIEVE and HP-PLOT Q) that allow for the separation of permanent gases (H_2_, CO, CO_2_, CH_4_) from CO_2_ and hydrocarbons between C2 and C6. Standard gas cylinders were used for the calibration of the equipment, which allowed the authors to obtain the composition of the gases in volumetric percentage. The calorific values of the gases were calculated based on their composition and the calorific value of each of their constituent substances.

### 2.5. Characterisation of Catalysts

#### 2.5.1. Chemical Analysis

The amounts of Ni, Co and Pd in the catalyst were determined by elemental inorganic chemical analysis. For this purpose, plasma spectroscopy analysis (ICP-AES, Perkin-Elmer Optima 2000, Waltham, MA, USA) was used. Prior to analysis, the catalysts were dissolved in a HF/HNO_3_/HCl = 3:3:2 mixture using a Milestone ETHOS 1 digester at 190 °C. The purities and brand names of the acids used were as follows: HF 48 wt%, EMSURER; HNO_3_ 65 wt%, Panreac; HCl 37 wt%, Panreac.

#### 2.5.2. Physical Adsorption/Desorption with Nitrogen

Physisorption with N_2_ allows us to know the surface properties of the catalyst, such as BET surface area, pore volume and mean pore diameter. For this purpose, the Autosorb 1C-TCD (Quantachrome, Boynton Beach, FL, USA) was used with the two catalysts. Before starting the N_2_ adsorption/desorption analysis, the samples were degassed at 150 °C for 12 h. The specific surface area was calculated using the Brunauer–Emmett–Teller (BET) method. The total pore volume and the mean pore diameter were estimated following the Barrett–Joyner–Halenda (BJH) method, while the micropore volume was calculated according to the Dubinin–Radushkevich (DR) method.

#### 2.5.3. Chemical Adsorption of CO

The active metal surface area (AMSA) and the metal dispersion were determined by chemical adsorption by CO in a Micromeritics^®^ AutoChem II apparatus (Norcross, GA, USA). The samples were reduced prior to analysis at 700 °C in the presence of a 5 vol% H_2_-Ar mixture. Subsequently, the CO-chemisorption capacity was measured at 35 °C in the presence of a 5 vol% CO-He mixture.

#### 2.5.4. Reduction at Programmed Temperature with H_2_

This technique was employed to determine the reduction temperature of the reducing species in the previously calcined catalysts. For this purpose, Micromeritics^®^ AutoChem II equipment (Norcross, GA, USA) with a thermal conductivity detector was used. A 5 vol% H_2_/Ar mixture was used with approximately 0.1 g of catalyst from room temperature to 950 °C, with a heating rate of 5 °C/min.

#### 2.5.5. Programmed Thermal Desorption with NH_3_

This technique was used to determine the acidity of the catalysts, previously calcined. The Micromeritics^®^ AutoChem II equipment was employed (Norcross, GA, USA) for the measurements. First, the samples were reduced at 700 °C with a 5 vol% H_2_/Ar mixture. After 30 min with He, it was cooled down to 100 °C, and NH_3_ adsorption was run for 30 min. Subsequently, the physically adsorbed NH_3_ was desorbed with He at 150 °C and the chemically adsorbed NH_3_ was desorbed between 150 °C and 900 °C, with a heating rate of 10 °C/min. The consistency of the acidity was differentiated as a function of the temperature required to desorb the adsorbed ammonia. In this way, three classes of acidity were differentiated: weak acidity below 250 °C, medium acidity between 250 °C and 450 °C and strong acidity between 450 °C and 900 °C. Once the signal emitted by the equipment was calibrated, the NH_3_ concentration was obtained in units of Ncm^3^/min. Subsequently, using the ideal gas equation and corrected with the mass of catalyst used in the analysis, the mmol_NH3_/(min·g) desorbed was obtained. Finally, by integrating over the desired time, adsorbed mmol_NH3_ per gram of catalyst were obtained.

#### 2.5.6. X-ray Diffraction

Qualitative and quantitative determination of the crystalline phases of the catalysts were carried out, identifying the crystalline compounds by X-ray diffraction. The equipment used was a Seifert XRD 3000P diffractometer (North Kingstown, RI, USA), with a PWBragge-Brentano q/2q 2200 goniometer, inclined monochromate graphite and automatic slot using Cu Kα radiation. Phase identification was performed using the Power Diffraction File database (PDF-2,4) and the International Centre for Diffraction Data (ICCD, Cambridge, UK). The mean particle size of the crystalline phases was calculated using the Scherrer equation.

## 3. Results and Discussion

In this section, the characterisation results of the two employed catalysts are given (Section 3.1) followed by the results of the pyrolysis experiments and the characterisation of the vapours and liquids generated (Section 3.2). The residual refractory brick employed as fixed bed material in the tubular reactor has been previously characterised and published by the authors, and it did not have catalytic properties [24].

### 3.1. Catalysts Characterisation

#### 3.1.1. Surface Area, Porosity and Chemical Analysis

The BET surface area, porosity and metallic quantities of the two catalysts used are presented in Table 1.

Both catalysts showed a low BET surface area, especially the olivine catalyst. In general, large surface areas are expected to promote contact between reactants and active sites. Therefore, surface area is often an important parameter in heterogeneous catalysis, which is often related to pore diameters. However, due to the relatively large size of the molecules produced during pyrolysis of thermosetting resins, surface area and pore diameter may not be critical parameters, as the produced molecules or coke may block small pores or cover the surface. Therefore, the size of the surface area may not be the most decisive parameter in determining the activity of a catalyst for the treatment of vapours generated in the pyrolysis process of end-of-life plastic materials. Ni commercial catalyst had a higher surface area (99 m^2^/g), with a significantly larger average pore diameter (163 Å) and larger pore volume. Finally, it could be said that the olivine-supported catalyst produced in the laboratory was not porous, as the few pores it contained were very small (35 Å).

As for the amount of metal in both catalysts, there were no significant differences between the nominal values expected of the deposited metal amounts (Ni, Co and Pd) and the real values determined using the ICP-AES technique.

#### 3.1.2. Active Surface and Metal Dispersion

Table 2 shows the catalyst active metal surface area (AMSA), metal dispersion and CO µmoles adsorbed per gram of sample determined on the fresh catalysts by CO chemisorption. The Ni commercial catalyst adsorbed a significant amount of CO, promoted by its high Ni content (39.3 wt%), with a higher active surface area and metal dispersion than the olivine-supported catalyst. The dispersion and active surface area of the Ni commercial catalyst were not as high as could be expected from its high nickel content, which may be because nickel accumulated on its low surface area to form large particles. In the case of the olivine-supported catalyst, it showed poor results. In addition, in this case, the amount of metal was high, but the BET surface area was very low, which meant that the large amount of metal was much accumulated, resulting in a very small dispersion. It should be noted that in the case of this catalyst, the dispersion results might not be very accurate, since when there is more than one metal, it is difficult to relate the amount of CO adsorbed with the specific interaction of each metal.

#### 3.1.3. Reduction Temperature

**Figure 1:**
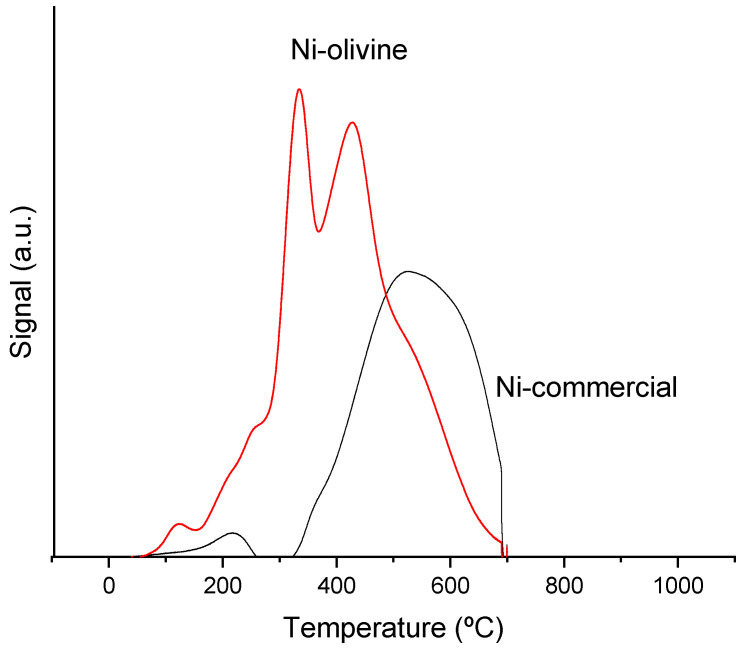
Temperature-Programmed Reduction (TPR) analysis of the catalysts.

Figure 1 shows the profiles of hydrogen consumed by the catalysts as a function of temperature, corrected by the mass of each catalyst and measured in arbitrary units. This signal intensity is proportional to the amount of hydrogen consumed during the reduction step required with the catalysts to reach the active phase of the metals for catalytic treatment of the pyrolysis vapours.

The Ni commercial catalyst showed the main reduction signal between 320 °C and 700 °C. The good metal dispersion observed in the previous section for this catalyst facilitated metal reduction. In the case of the Ni–olivine catalyst, two reduction peaks could be differentiated. The initial reduction peak might be a consequence of the reduction of Fe_2_O_3_ and MgFe_2_O_4_ compounds in the olivine composition itself [25], while the second peak could be due to Ni reduction. This Ni in this catalyst was forming large particles (as seen in the previous section), which may have hindered the reduction, since hydrogen is not easily penetrated to allow for the continuation of reducing Ni atoms.

#### 3.1.4. Crystallinity and Crystalline Particle Size

**Figure 2:**
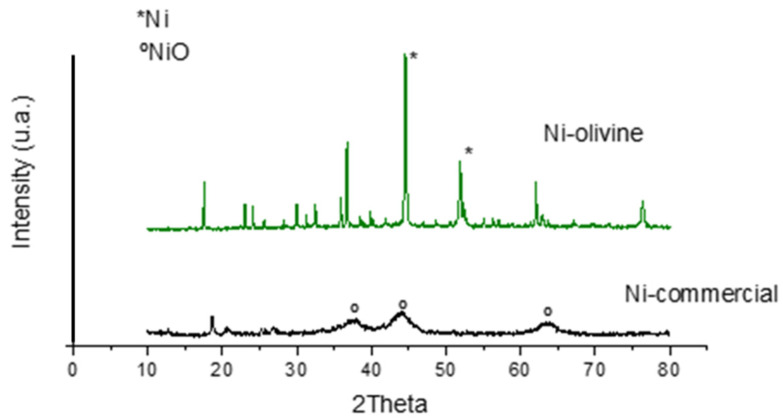
XRD diffractograms of the catalysts.

The X-ray diffractograms obtained for Ni–olivine and Ni commercial catalysts are shown in Figure 2. In the Ni–olivine catalyst, metallic nickel peaks were observed at values of 2θ = 45° and 2θ = 52° (Ni, 01-087-0712, identified in the picture with the symbol *) together with signals from the support itself (Mg_2_SiO_4_, 01-084-1402). There was no trace of Pd or Co metals. Metallic nickel and cobalt have a very similar diffractogram, so nickel probably covered the signal of cobalt, making it difficult to differentiate the signal of each [17]. In the case of palladium, it is likely to be found in very small crystals (<1 nm), making it impossible to detect the diffraction peaks by XRD.

In the case of the Ni commercial catalyst, the peaks of the NiO compound were observed at the values 2θ = 38°, 2θ = 44° and 2θ = 63° (00-022-1189, identified in the image with the symbol °). Metallic Ni peaks were not detected on the diffractogram, indicating that a quick oxidation of the surface during handling of the sample could have occurred prior to XRD analysis.

This technique also allows for the average crystal size to be calculated using the Scherrer equation. The Ni commercial catalyst had particles smaller than 5 nm (NiO), while the Ni–olivine had crystalline particles with an average size of 150 nm. This large crystalline particle size was expected in view of the large amount of metal in this catalyst and the low metal dispersion due to the small surface area. In contrast, the commercial Ni catalyst showed a very small crystalline particle size.

#### 3.1.5. Acidity

**Figure 3:**
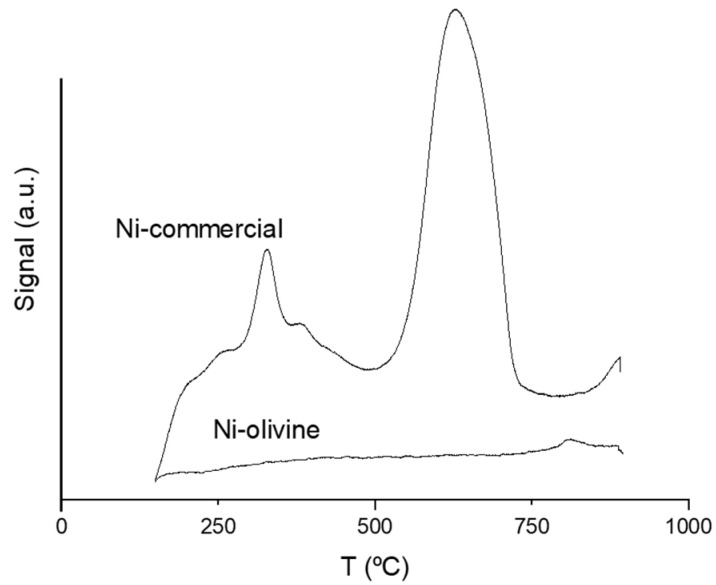
Acidity results of the catalysts by NH_3_-TPD.

Figure 3 shows the mass-corrected acidity intensity of the catalysts in arbitrary units, showing the desorption profiles of the previously adsorbed ammonia as a function of temperature. Observing the image, it can be stated that the Ni commercial catalyst was an acid catalyst, with a main acidity zone in the range 500–750 °C, while the olivine-based one did not show acidity.

Table 3 shows the total acidity of the catalysts (mmol_NH3_/g) and the acidity strength (obtained as a function of the temperature required to desorb the adsorbed ammonia). In this case, three classes of acidity were differentiated: weak acidity below 250 °C, medium acidity between 250 °C and 450 °C, and strong acidity between 450 °C and 900 °C. The olivine catalyst has not been included in the table, since, as previously indicated, it did not show acidity. The Ni commercial catalyst was quite acidic and it was very clearly observed that its main type of acidity was strong, corresponding to ammonia desorbed at high temperatures.

### 3.2. Pyrolysis Results

The pyrolysis conditions tested will be analysed and discussed in this section. In every experiment, the batch reactor was heated up to 500 °C with no N_2_ flow fed into the pilot plant. The operating conditions were defined based on different parameters set in the tubular reactor that was placed in series with the pyrolysis reactor. In Table 4, the main operating conditions of the seven experiments, the yields and the composition of the obtained gas phase are shown. First of all, experiments without vapour treatment (corresponding to column 1) are indicated; then, the effect of thermal treatment is given (700 °C, 800 °C, 900 °C), and later, the results of thermo-catalytic treatment are shown (700 °C commercial, 700 °C olivine, 800 °C commercial). In Table 5, a thorough chemical characterisation of the collected liquids is given, together with their appearance after mixing the condensate collected in the three condensation flasks. For each experiment, the amount of water obtained (area%), the amount of identified organic compounds (area%) and the amount of unidentified compounds (area%) are given. In the group “Not identified”, compounds defined with insufficient quality (<85%) in the mass spectrometer detector database have been grouped together, and “-“ has been used for non-detected compounds. Within the identified organic compounds, all detected compounds have been listed explicitly.

The discussion of the results will be focused on the amount of gas and liquid produced in the pyrolysis experiments (yields), in the composition of the gas phase (main interest being H_2_ and syngas (H_2_+CO)) and in the composition of the liquid phase (main interest being to recover any compound and/or to be not hazardous).

#### 3.2.1. Influence of Thermal Treatment in Pyrolysis Vapours

In order to see the necessity of pyrolysis vapour treatment, an initial analysis of the results obtained in the absence of the tubular reactor (first column of Table 4, “No” test) is required. The solid yield in all the experiments was very similar (≈80 wt%), which was expected, as the operating conditions in the pyrolysis reactor were the same (heating up to 500 °C, at 3 °C/min and no N_2_ fed). The main critical part of not employing the treatment of the vapours was the gas and liquid compositions (Table 4 and Table 5). The gas produced without the tubular reactor was composed of 97 vol% of CO. The liquid had a very complex composition, with 34.5 area% of water and 54.4 area% of identified organic compounds, which were 25 different compounds. Among these 25 identified compounds, there were nitrogenous organic compounds, oxygenated organic compounds, sulphur organic compounds and polycyclic aromatic hydrocarbons, giving hazardous properties to this stream. In fact, in the experiment without the tubular reactor, in the collected liquids, organic and aqueous phases were observed, while in the experiment with the tubular reactor, only the aqueous phase was obtained. Therefore, the liquid condensed in the pyrolysis process without vapour treatment should be properly collected, stored and managed as waste, with an economic and environmental cost.

In order to analyse the effect of the thermal treatment on pyrolysis vapours, the results of the three tests carried out at 700 °C, 800 °C and 900 °C (temperature of the tubular reactor) are going to be discussed. Regarding the condensate yields, as the temperature increased, a decrease in liquid yield was observed (17.0 wt% (700 °C) > 15.4 wt% (800 °C) > 13.3 wt% (900 °C)). In fact, as the temperature increased, the vapour cracking efficiency of the tubular reactor increased, producing compounds with higher vapour pressure, thus increasing the gaseous yield (3.1 wt% (700 °C) < 4.8 wt% (800 °C) < 6.8 wt% (900 °C)). As can be seen in the pictures of the liquids in Table 5, the higher the temperature in the tubular reactor, the higher the transparency and the lower the viscosity.

Comparing the composition of the gases produced in these three tests, at the lowest temperature (700 °C), the highest amount of H_2_ (91.7 vol%) was produced. The point is that high temperatures can disfavour the thermodynamics of some exothermic reactions. One of the disfavoured reaction media could be the Water Gas Shift (CO + H_2_O ←→ CO_2_ + H_2_). In this case, the reaction will be favoured to the left, decreasing H_2_ and increasing CO in the products. On the other hand, from the appearance of the collected liquids, the presence of hydrocarbons in the liquids obtained in the 700 °C test can be predicted. Therefore, it could be concluded that a temperature of 700 °C was not sufficient to break down the organic compounds that have been collected as condensates. This is why in the 700 °C test, the lowest amount of CO (8.0 vol% (700 °C) < 20.6 vol% (800 °C) < 20.5 vol% (900 °C)) was obtained in the gases. The same trend was also observed for CH_4_: the lowest amount of methane (0.3 vol% (700 °C) < 4.8 vol% (800 °C) ~ 4.3 vol% (900 °C)) was obtained in the test with the lowest temperature in the tubular reactor. Therefore, it can be suspected that the expected steam reforming in the tubular reactor had not been very efficient. With a temperature of 800 °C in the tubular reactor, thermal cracking and steam reforming were favoured, resulting in an increase in CH_4_ (4.8 vol%) and CO (20.6 vol%). Due to the increased production of these compounds, the volume percentage of H_2_ decreased to 74.4 vol%. However, 95.0 vol% of the gas was synthesis gas (H_2_+CO), which is of great industrial applicability. Between the 800 °C test and the 900 °C test, there were no major differences in the gas composition, but there were differences in the amounts of gas produced (4.8 wt% (800 °C) < 6.8 wt% (900 °C)).

Concerning the effect of the thermal treatment in the liquids, it can be seen that a higher amount of water was produced using the tubular reactor at increasing temperature (45.2 area% (700 °C) < 46.8 area% (800 °C) < 92.1 area% (900 °C)). The identified organic compounds, except in the 700 °C test, decreased with increasing temperature (43.6 area% (800 °C) > 4.3 area% (900 °C)). In fact, in the 700 °C test, the percentage of “Unidentified” compounds was quite high compared to other thermal treatments (14.6 area% (700 °C) > 9.6 area% (800 °C) > 3.6% (900 °C). With increasing temperature in the tubular reactor, several organic compounds represented in the “Without tubular reactor” test were no longer detected, because more thermal cracking of the pyrolysis vapours occurred. Consequently, there was less aniline (30.2 area% (700 °C) > 26.2 area% (800 °C) > 1.3 area% (900 °C)) and phenol (6.2 area% (700 °C) > 2.8 area% (800 °C) > 1.2 area% (900 °C)) in the identified organic compounds. Therefore, because the production of organic compounds decreased considerably in the tests using the thermal treatment of pyrolysis vapours, although the quantity of water produced was not very high, the percentage of water increased. In fact, high temperatures favoured the breaking of the bonds of the biggest liquid organic molecules, such as aniline. In this context, the CH_4_, H_2_, CO and CO_2_ produced from the cracking reactions in the tubular reactor had an optimal situation in the presence of water vapour to initiate reforming or Water Gas Shift reaction pathways (CO + H_2_O ←→ CO_2_ + H_2_).

Taking into account the previous discussions, the smaller quantity of liquids obtained with a simplified composition, together with an interesting composition of the gas obtained (95.5 vol% of syngas), it can be concluded that the best vapour thermal treatment was the one carried out at 900 °C.

#### 3.2.2. Catalytic Treatment of Pyrolysis Vapours

Given that operating at 900 °C in the tubular reactor requires high energy consumption, in this section, the effect of incorporating catalysts has been studied with the aim of achieving a similar quality of gas and liquid products but at a lower temperature. Two different catalysts have been used at 700 °C (700 °C commercial and 700 °C olivine) and the Ni commercial catalyst has also been tested at 800 °C (800 °C commercial). As a reference, the experiments carried out at 700 °C will be taken as a reference for comparison of the results. As can be seen in Table 4, the presence of Ni commercial and Ni–olivine catalysts decreased the condensate yields (17.0 wt% (700 °C) > 16.1 wt% (700 °C olivine) > 15.4 wt% (700 °C commercial). When Ni commercial and Ni–olivine catalysts were used, the gaseous yield was increased (3.1 wt% (700 °C) < 3.7 wt% (700 °C olivine) < 4.3 wt% (700 °C commercial)). As can be seen, the highest gaseous yield was achieved with the Ni commercial catalyst. Although both catalysts contained high amounts of Ni, the Ni commercial catalyst was the one with the highest Ni percentage and the highest active surface, as well as showing an acidic support, as discussed in Section 3.1. Therefore, it could be concluded that it may had influenced the reforming reactions on the one hand and the cracking of the vapours on the other hand. Moreover, it should be taken into account that in this type of reaction, some authors underline that the efficiency of catalysts supported on alumina could be higher [26,27]. This is in accordance with the results of the Ni commercial catalyst, with CaO/Al_2_O_3_ as the support, where less condensates and more gases were obtained.

As for the gas composition, comparing the gas compositions of the Ni commercial and Ni–olivine tests, with only thermal treatment (700 °C), it is observed that an increase in the amount of H_2_ was achieved in both cases (91.7 vol% (700 °C) < 93.1 vol% (700 °C commercial) < 94.8 vol% (700 °C olivine)). CO followed, although its value was lower than that obtained in the thermal treatment in both cases (8.0 vol% (700 °C) > 5.9 vol% (700 °C commercial) > 5.2 vol% (700 °C olivine)). In view of the results, both in the Ni commercial case and in the Ni–olivine case carried out in the laboratory, the presence of Ni promoted dry reforming (C_n_H_m_ + nCO ←→ 2nCO + m/nH_2_) and wet reforming (C_n_H_m_ + nH_2_O ←→ nCO + (n + m/s)H_2_). Therefore, in these two cases, in addition to no trace of CO_2_ being observed in the test, the appearance of the collected liquids was more transparent than in the case where only thermal treatment had been implemented. In the case of the Ni commercial catalyst, due to its acidity, it can also be predicted to promote cracking reactions (pC_x_H_y_ ←→ qC_n_H_m_ + rH_2_), as well as the effect of cobalt in the Ni–olivine catalyst, which can promote dehydrogenation reactions [28]. Therefore, it can be said that some compounds that could not be cracked by thermal treatment alone were cracked thanks to the presence of the catalysts. In the test using Ni–olivine, although the difference was not high, an increase in H_2_ was achieved (94.8 vol%). In fact, it is important to remember that this catalyst had Ni, Co and Pd as metals. Cobalt may have been able to break C-C bonds and palladium to increase the stability of the catalyst, preventing the tendency of nickel to produce coke [17,18,19,28,29]. As for the calorific value, the HHV of the gas obtained in the three tests compared in this section was very similar (11.7–11.9 MJ/m^3^).

Regarding the collected liquids, the presence of the catalyst increased the amount of water (45.2 area% (700 °C) < 58.4 area% (700 °C olivine) < 74.7 area% (700 °C commercial)). On the other hand, the number of organic compounds identified in the catalytic tests was reduced from 25 compounds defined in the test without the tubular reactor to a maximum of 6 with the catalytic treatment.

In the experiments using Ni commercial, the increase in water (74.7 area% (700 °C commercial) > 45.2 area% (700 °C), decrease in aniline (19.1 area% (700 °C commercial) < 30.2 area% (700 °C)) and decrease in phenol (2.4 area% (700 °C commercial) < 6.2 area% (700 °C)) was noteworthy in the collected liquids. In addition, some isoquinoline (1.2 area%) was generated. In total, 97.4% of the chromatogram area was identified as compounds, and 2.6% was unidentified. In other words, it seems that this catalyst was effective in the reduction of organic compounds in the liquid phase thanks to the aforementioned reaction pathways, mainly cracking and reforming, greatly simplifying the composition of the condensed phase.

With regard to the “700 °C-olivine” test, there was a presence of organic compounds in the liquids, such as toluene (2.2 area%), 2-methyl-benzofuran (0.9 area%), aniline (14.9 area%), phenol (6.1 area%), styrene (0.4 area%) and benzofuran (1.6 area%). In other words, a decrease in organic compounds was observed with respect to the thermal test (water percentage of 58.4 area%), but the effect was not as significant as with the commercial Ni.

Analysing the previously described results of Table 4 and Table 5, the experiment at 700 °C with the Ni commercial catalyst was selected as the best among the thermo-catalytic treatments. This was decided taking into account the high quantity of hydrogen in gases (93.1 vol%), the low amount of liquid obtained in this test (15.4 wt%), and the simple composition of this liquid, being almost an aqueous aniline solution. The aniline can be useful for the chemical industry (dyes, antioxidants, pharmaceuticals and rubber industries) [30]. However, if aniline could not be used industrially, it must be taken into account that these liquids should be managed as process waste. From a waste management point of view, the liquids obtained in the 900 °C test could be more attractive, as the liquid fraction produced is smaller. In order to analyse whether it was possible to improve the liquid composition of the “700 °C-commercial” test, an experiment at 800 °C with an Ni commercial catalyst was carried out.

Regarding the condensed liquid yields, in the experiment at 800 °C with the Ni commercial catalyst, an improvement was observed, i.e., the amount of liquid obtained with “700 °C-commercial” was reduced (15.4 wt% (700 °C commercial) > 13.6 wt% (800 °C commercial)) and was very close to the amount achieved at 900 °C (15.4 wt% (700 °C commercial) > 13.3 wt% (900 °C). Also noteworthy was the decrease in condensed liquids (15.4 wt% (700 °C commercial) > 13.6 wt% (800 °C commercial) > 13.3 wt% (900 °C)), with the minimum reached at 900 °C. Moreover, the appearance of these liquids was the most transparent among those obtained in all the tests.

The gaseous yield of the 800 °C Ni commercial experiment was improved, i.e., the quantity obtained at 700 °C with the Ni commercial catalyst (4.3 wt% (700 °C commercial) < 6.4 wt% (800 °C commercial)) was increased and was very close to the quantity obtained at 900 °C (6.4 wt% (800 °C commercial) < 6.8 wt% (900 °C)). The gas composition obtained at 900 °C with thermal treatment was higher for H_2_ than that obtained at 800 °C with the Ni commercial catalyst (72.3 vol% (800 °C commercial) < 75.0 vol% (900 °C)), smaller for CO (20.9 vol% (800 °C commercial) > 20.5 vol% (900 °C)) and smaller for CH_4_ (3.7 vol% (800 °C commercial) < 4.3 vol% (900 °C)). In the case of CO_2_, the use of the catalyst lead to a higher CO_2_ production (3.1 vol% (800 °C commercial > 0.2 vol% (900 °C). In fact, less coke was experimentally observed in the reactor, so it was assumed that there was a lower probability that the Boudouard reaction (C + CO_2_ ←→ 2CO) will take place, with a lower CO_2_ consumption, thus increasing the presence of CO_2_ in the gaseous composition. Compared to the experiment at 700 °C with the Ni commercial catalyst, it was observed that with an increasing temperature, the appearance of the collected liquid had greatly improved. Furthermore, with an increase in temperature to 800 °C, the HHV of the gases increased (11.9 MJ/Nm^3^ (700 °C commercial) < 12.2 MJ/Nm^3^ (800 °C commercial)).

Looking at Table 5 and analysing the “900 °C”, “700 °C-commercial” and “800 °C-commercial” experiments, it can be said that when Ni commercial was used in the tubular reactor, increasing its temperature to 800 °C, the amount of water increased a lot, generating a greater quantity than in the 900 °C test (97.5 area% (800 °C commercial) > 92.1 area% (900 °C) > 58.7 area% (700 °C commercial)). In addition, the organic compounds identified practically disappeared (0.9 area% aniline, 0.6 area% quinoline and 0.5 area% 6-methyl-quinoline). Therefore, the total compounds identified accounted for 99.5 area%, while among those identified, 97.5 area% corresponded to water. In view of these results, it is evident that the Ni commercial catalyst allowed reducing the temperature of the tubular reactor by 100 °C, with a consequent reduction in energy consumption, and it has also been possible to improve the quality of the liquids from the point of view of their management. In summary, among the thermal and thermo-catalytic treatments of pyrolysis vapours carried out, the most interesting results were obtained at 800 °C and with the commercial Ni catalyst: lower production of collected liquid (13.6 wt%), high amount of gas (6.4 wt%) and 93.2 vol% of synthesis gas in the gas.

## 4. Conclusions

The main conclusions obtained in the optimisation of the pyrolysis vapour treatment described above are summarised as follows:

- The problem that remains to be solved in the pyrolysis treatment of fibre-reinforced plastic is mainly the liquid stream.
- In order to obtain useful liquids and gases, it is necessary to place a thermal or thermo-catalytic vapour treatment reactor in series with the pyrolysis reactor.
- Among the thermal treatments of pyrolysis vapours, the most interesting results have been obtained at 900 °C: 95.5 vol% of synthesis gas, 75.0 vol% of H_2_ and 92.1 area% of the collected liquid being water.
- The main effect of the catalysts have been on the composition of the liquids, not so much on the yields.
- Among the thermo-catalytic treatments of pyrolysis vapours, the most interesting results have been obtained at 800 °C with the Ni commercial catalyst: 93.2 vol% of synthesis gas, 72.3 vol% of H_2_ and 97.5 area% of the collected liquids being water.
- It is proposed that the best experimental conditions are with the tubular reactor at 700 °C with the Ni commercial catalyst, as long as aniline can be recovered from the liquid and the gas is 93.1 vol% H_2_. If aniline from the liquid phase cannot be recovered and used, we propose as the best experimental conditions the tubular reactor at 800 °C with the Ni commercial catalyst, because the liquid is almost entirely water, so it can be an inert stream to be managed without economic or environmental cost. At these conditions, the obtained gas flow will have a 72.3 vol% of H_2_ and a 93.2 vol% of syngas.

## Figures and Tables

**Table 1:** Physic-chemical characteristics of the catalysts.

Catalyst	BET (m^2^/g)	Pore Volume (cm^3^/g)	Average Pore Diameter (Å)	Metal (wt%)		
				Ni	Pd	Co
Ni commercial	99	0.404	163	39.3	-	-
Ni–olivine	4	0.016	35	29.9	0.8	8.6

**Table 2:** Active metal surface area (AMSA), metal dispersion (MD) and total adsorbed CO of the catalysts.

Catalyst	AMSA (m^2^/g)	MD (%)	Total Adsorbed CO (µmol/g_sample_)
Ni commercial	0.85	0.32	21.7
Ni–olivine	0.02	0.01	0.5

**Table 3:** Results of Ni commercial catalyst acidity as a function of strength: weak (<250 °C), medium (250–450 °C) and strong (450–900 °C).

Catalyst	Total Acidity (mmol_NH3_/g)	Weak Acidity (mmol_NH3_/g)	Medium Acidity (mmol_NH3_/g)	Strong Acidity (mmol_NH3_/g)
Ni commercial	0.850	0.071	0.239	0.540

**Table 4:** Pyrolysis experiments carried out, indicating the main operating parameters, yields (wt%), gas composition and the appearance of the collected liquids.

Tubular Reactor	No	Yes					
T tubular reactor (°C)	-	700	800	900	700	700	800
Catalyst	-	-	-	-	Ni commercial	Ni–olivine	Ni commercial
Solid (wt%)	80.7	79.9	79.8	79.9	80.3	80.2	80.0
Liquid (wt%)	16.3	17.0	15.4	13.3	15.4	16.1	13.6
Gas ^1^ (wt%)	3.0	3.1	4.8	6.8	4.3	3.7	6.4
Gases composition (vol%) and HHV (MJ/m^3^N)							
H_2_	0.0	91.7	74.4	75.0	93.1	94.8	72.3
CO	97.0	8.0	20.6	20.5	5.9	5.2	20.9
CO_2_	3.0	<0.1	0.2	0.2	<0.1	<0.1	3.1
CH_4_	0.0	0.3	4.8	4.3	1.0	<0.1	3.7
HHV (MJ/m^3^ in N.C.)	11.2	11.8	12.9	12.7	11.9	11.7	12.2
Collected liquids							

^1^ By difference.

**Table 5:** Composition, in area%, of the pyrolysis liquids collected in the different experiments carried out. Compounds not detected are indicated with “-”.

Tubular Reactor	No	Yes					
T tubular reactor (°C)	-	700	800	900	700	700	800
Catalyst	-	-	-	-	Ni commercial	Ni–olivine	Ni commercial
Water (area%)	34.5	45.2	46.8	92.1	74.7	58.4	97.5
Identified organic compounds (area%)	54.4	40.2	43.6	4.3	22.7	36.1	2.0
Identified in total (area%)	88.9	85.4	90.4	96.4	97.4	94.5	99.5
Unidentified (area%)	11.1	14.6	9.6	3.6	2.6	5.5	0.5
Collected liquids	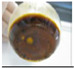	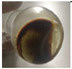	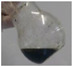	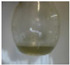	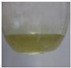	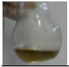	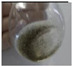
Identified organic compounds list (area%)							
Toluene (C_7_H_8_)	2.1	-	-	-	-	2.2	-
Acetone (C_3_H_6_O)	1.6	-	-	-	-	-	-
N,N-dimethylbenzamide (C_8_H_11_N)	0.4	-	-	-	-	-	-
2-methylbenzofuran (C_9_H_8_O)	0.6	1.1	-	-	-	0.9	-
N-methylaniline (C_7_H_9_N)	1.5	-	-	-	-	-	-
Aniline (C_6_H_7_N)	11.6	30.2	26.2	1.3	19.1	14.9	0.9
1-phenyl-1H-pyrrole (C_10_H_9_N)	0.4	-	-	-	-	-	-
Quinoline (C_6_H_9_N_3_O_2_)	1.0	-	0.5	-	-	-	0.6
2-methylphenol (C_7_H_8_O)	2.7	-	-	-	-	-	-
Phenol (C_6_H_6_O)	10.9	6.2	2.8	1.3	2.4	6.1	-
Diphenylether (C_12_H_10_O)	3.8	-	-	-	-	-	-
6-methylquinoline (C_10_H_9_N)	0.5	-	-	-	-	-	0.5
2-ethylphenol (C_8_H_10_O)	0.8	-	-	-	-	-	-
4-methylphenol (C_7_H_8_O)	2.2	-	-	-	-	-	-
Caprolactam (C_6_H_11_NO)	10.1	-	-	-	-	-	-
9H-Xanthene (C_13_H_10_O)	0.5	-	-	-	-	-	-
Diphenyl disulphide (C_12_H_10_S_2_)	1.3	-	-	-	-	-	-
Ethylbenzene (C_8_H_10_)	0.4	-	-	-	-	-	-
1,3-dimethylbenzene (C_8_H_10_)	0.2	-	-	-	-	-	-
Methylthiobenzene (C_7_H_8_S)	0.3	-	-	-	-	-	-
p-aminotoluene (C_7_H_9_N)	0.3	-	-	-	-	-	-
2,6-dimethylphenol (C_8_H_10_O)	0.3	-	-	-	-	-	-
2-1-penthylfuran (E), (C_9_H_12_O)	0.3	-	-	-	-	-	-
4-ethylphenol (C_8_H_10_O)	0.3	-	-	-	-	-	-
Diphenyl sulphide (C_12_H_10_S)	0.3	-	-	-	-	-	-
Styrene (C_8_H_8_)	-	0.6	-	-	-	0.4	-
Benzonitrile (C_7_H_5_N)	-	2.1	2.8	-	-	-	-
Benzene (C_6_H_6_)	-	-	4.6	-	-	-	-
Naphthalene (C_10_H_8_)	-	-	6.0	-	-	-	-
2-ethylnaphthalene (C_12_H_10_)	-	-	0.7	-	-	-	-
Isoquinoline (C_9_H_7_N)	-	-	-	1.7	1.2	-	-
Benzofuran (C_8_H_6_O)	-	-	-	-	-	1.6	-

## Data Availability

Data are contained within the article.
